# A Bayesian Model of the Uncanny Valley Effect for Explaining the Effects of Therapeutic Robots in Autism Spectrum Disorder

**DOI:** 10.1371/journal.pone.0138642

**Published:** 2015-09-21

**Authors:** Yuki Ueyama

**Affiliations:** Department of Rehabilitation Engineering, Research Institute of National Rehabilitation Center for Persons with Disabilities, Tokorozawa, Saitama, Japan; University of New South Wales, AUSTRALIA

## Abstract

One of the core features of autism spectrum disorder (ASD) is impaired reciprocal social interaction, especially in processing emotional information. Social robots are used to encourage children with ASD to take the initiative and to interact with the robotic tools to stimulate emotional responses. However, the existing evidence is limited by poor trial designs. The purpose of this study was to provide computational evidence in support of robot-assisted therapy for children with ASD. We thus propose an emotional model of ASD that adapts a Bayesian model of the uncanny valley effect, which holds that a human-looking robot can provoke repulsion and sensations of eeriness. Based on the unique emotional responses of children with ASD to the robots, we postulate that ASD induces a unique emotional response curve, more like a cliff than a valley. Thus, we performed numerical simulations of robot-assisted therapy to evaluate its effects. The results showed that, although a stimulus fell into the uncanny valley in the typical condition, it was effective at avoiding the uncanny cliff in the ASD condition. Consequently, individuals with ASD may find it more comfortable, and may modify their emotional response, if the robots look like deformed humans, even if they appear “creepy” to typical individuals. Therefore, we suggest that our model explains the effects of robot-assisted therapy in children with ASD and that human-looking robots may have potential advantages for improving social interactions in ASD.

## Introduction

According to the DSM-5, which is the most widely accepted classification of mental disorders, autism spectrum disorder (ASD) is behaviorally defined as a group of disorders with abnormal or impaired development in two areas: persistent deficits in social communication and social interaction, and restricted, repetitive patterns of behavior, interests, or activities [1]. Individuals with ASD experience the world and human behavior differently compared with typically developing individuals, as they react in an atypical way to stimuli [2]. There has been interest in using robots for intervention with children with ASD because robots can generate a high degree of motivation and engagement in children with learning disabilities and can be used to communicate, interact, display and recognize emotion, develop social competency, and maintain social relationships [3, 4]. Studies have shown that children with ASD tend to express more interest in robots than in non-robotic toys or human partners [5, 6]. Therefore, the use of robots as therapy tools may improve the engagement of children with ASD and elicit novel social behaviors [7, 8]. Additionally, the particular design used to create such robots may play a large role in helping children with ASD become more social [9].

**Fig 1:**
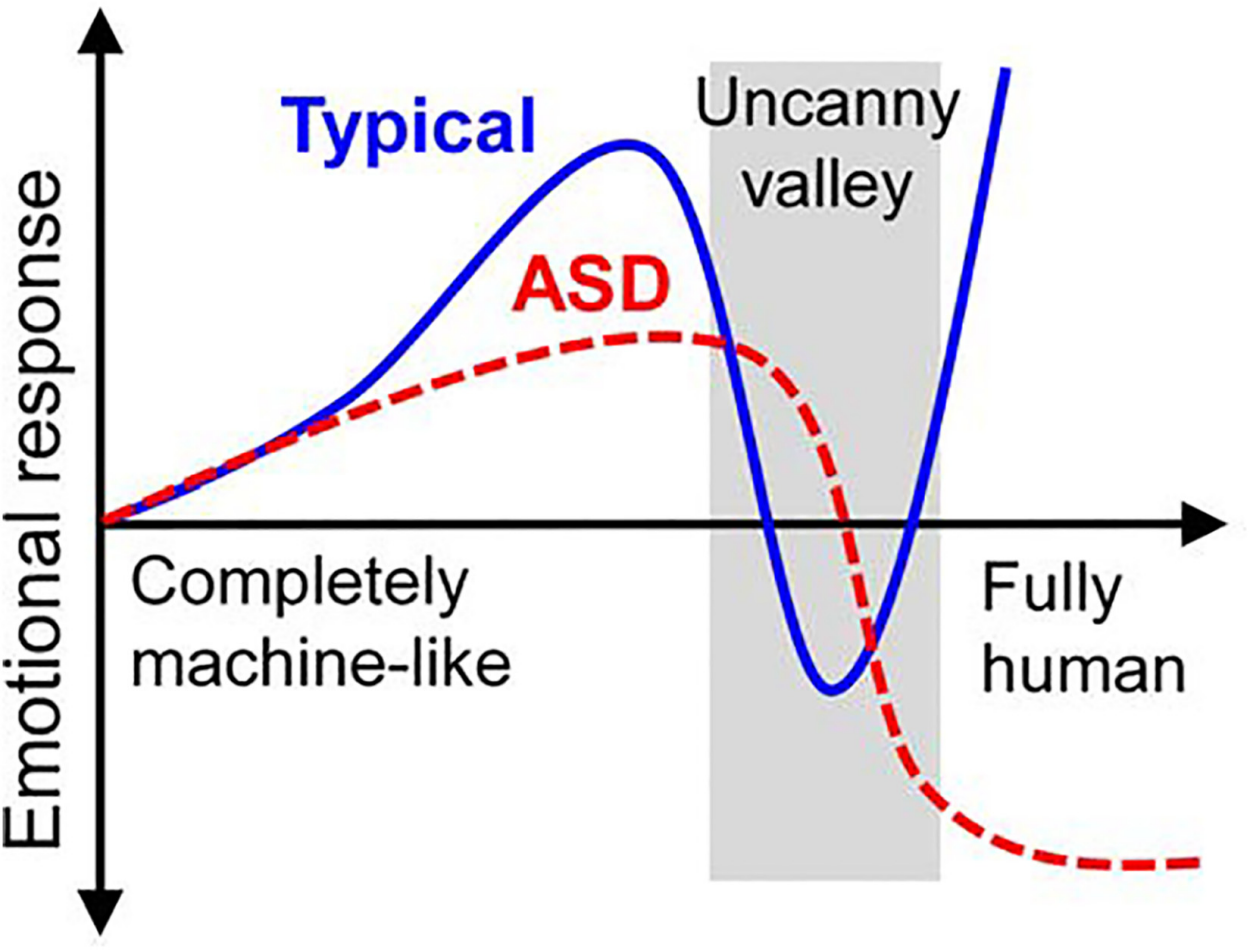
The uncanny valley hypothesis and the model’s prediction. The solid blue line indicates the uncanny valley effect. The red dashed line shows the model’s prediction for children with ASD.

When designing robot systems that interact with people, it is important to take into account the uncanny valley theory [10, 11]. This holds that, although cartoonish or other abstract human figures may elicit an immediate sense of familiarity in human observers, robots or animations that appear very similar, but not identical to, humans may trigger a sense of uneasiness [12]. This feeling is represented graphically by a sharp dip in familiarity, called the uncanny valley, in which the observer’s emotional response to the artificial character becomes drastically less positive (Fig 1, Typical). These unsettling emotions are thought to have an evolutionary origin, a theory that is supported by studies of monkeys [13] and human infants [14, 15]. In addition, an fMRI study reported that there is activation related to the uncanny valley in the human brain [16].

To avoid the uncanny valley, designers often create robots that do not resemble humans. For example, many therapeutic robots are designed to look like animals or to be cute and non-threatening, such as PARO [17, 18]. However, although a therapeutic robot called KASPER may closely resemble a human and seem creepy to a typical person, children with ASD have responded well to it [19]. In another study that used a robotic face as stimuli, children with ASD showed no increase in heart rate when exposed to a creepy face, whereas children without ASD appeared uncomfortable and exhibited an increased heart rate [20]. Additionally, the majority of children with ASD showed an increase in social communication when they interacted with a robotic face [21]. These studies suggest that children with ASD do not find human-looking robots creepy, although they feel uncomfortable interacting with most people. We thus postulated that ASD induces a unique emotional response curve more like a cliff than a valley (Fig 1, ASD). Given this, individuals with ASD may identify humans utilizing different neural systems and information to typical individuals [22–24], thus leading them to classify humans into different categories, compared to typical individuals.

The purpose of this study was to provide computational evidence for robot-assisted therapy for children with ASD. We hypothesize that: 1) individuals with ASD categorize humanness abnormally, and 2) their social interaction, defined as their emotional response to humans, may be improved by learning to categorize humans in the same manner as non-ASD sufferers. Based on these hypotheses, we modeled the emotional responses in ASD as the “uncanny cliff”, adapting a Bayesian model of the uncanny valley [25], and carried out numerical simulations of robot-assisted therapy to evaluate the effects. The results showed that even if a stimulus fell into the uncanny valley in the typical condition, it sometimes improved the emotional response in the ASD condition. Therefore, we concluded that our model may explain the effects of robot-assisted therapy in children with ASD, and that human-looking robots may have the potential to improve social interaction in ASD.

## Methods

### The uncanny valley model

A dimension of human-likeness can be defined in the uncanny valley theory as a smooth linear change in the degree of physical human-like similarity. The perceived human-likeness of objects can therefore be explained in terms of the psychological effect of categorical perception (CP) [26, 27]. CP is the experience of percept invariance in sensory phenomena that can be varied along a continuum. CP is related to how neural networks in the human brain detect the features of objects in the world, allowing them to be separated into categories based on perceived similarities and differences [28].

Feldman developed a computational model of the influence of CP, known as the perceptual magnet effect, using a Bayesian model of optimal statistical inference [29]. The perceptual magnet effect is a phenomenon that affects perceptual categorization; it has mainly been described with respect to vowels, and is characterized by a warping of perceptual space near phonemic category centers [30]. The effect makes people judge stimuli that are close to a category boundary as more dissimilar than stimuli that are distant from a category.

Recently, Moore proposed a model of the uncanny valley effect that is an extension of Feldman’s model [25]. This model could predict the differential perception distortion resulting from stimuli involving conflicting cues that induce perceptual tension at category boundaries, and it could explain the uncanny valley effect. According to Moore’s model, the distortion resulting from the perceptual magnet effect can be described by the following displacement function:

$$D(S_{i})=E(H|S_{i})-S_{i},$$ (1)

where *E*(*H* | *S*_*i*_) is the expected value of the perceptual humanness *H* given a physical stimulus *S*_*i*_ (*i* = 1, 2, …), which is a component of the multi-dimensional perceived stimuli *S*_1_, *S*_2_, …. The term *D*(*S*_*i*_) expresses a measure of perceptual distortion towards or away from the different categories. If *D*(*S*_*i*_) is positive, it represents a distortion in one direction in line with the stimulus axis. In contrast, a negative value of *D*(*S*_*i*_) represents a distortion in the opposite direction. Suppose a given category *C*_*j*_ (*j* = 1, 2, …), *E*[*H* | *S*_*i*_] is described as

$$E[H|S_{i}]=\sum_{j}P(C_{j}|S_{i})\frac{\sigma_{c_{j}}^{2}S_{i}+\sigma_{s_{i}}^{2}\mu_{c_{j}}}{\sigma_{c_{j}}^{2}+\sigma_{s_{i}}^{2}},$$ (2)

where *μ*_*cj*_ and *σ*_*cj*_ are the mean and standard deviations of the category, respectively, and *σ*_*si*_ is the measure of uncertainty related to the stimulus. Then, according to Bayes’ theorem, the posterior probability *P*(*C*_*j*_ | *S*_*i*_) is represented as

$$P(C_{j}|S_{i})=\frac{P(S_{i}|C_{j})\cdot P(C_{j})}{P(S_{i})},$$ (3)

where

$$P(S_{i})=\sum_{j}P(S_{i}|C_{j})\cdot P(C_{j}).$$

In the following, the posterior probability *P*(*S*_*i*_ | *C*_*j*_) is supposed using a Gaussian distribution:

$$P(S_{i}|C_{j})=N(\mu_{cj},\sigma_{c_{j}}^{2}+\sigma_{s_{i}}^{2}).$$

Imagine a situation in which there are multiple dimensions, and in which stimuli are perceived as multiple cues. In this case, Moore defined any differential perpetual distortion as the variance of the displacement values:

$$V=E\left[D{\left(S_{i}\right)}^{2}\right]-{\left(E\left[D(S_{i})\right]\right)}^{2}.$$ (4)

Here, *V* indicates the amount of perceptual tension, which would be a result of differential distortions between conflicting perceptual cues, such that *V* increases with greater perceptual conflict. Therefore, the emotional response function *F*(*S*_*i*_) can be provided by subtracting *V* from *P*(*S*_*i*_):

$$F(S_{i})=P(S_{i})-\alpha \cdot V,$$ (5)

where *α* is the scaling parameter set to 150, which reflects the sensitivity of an observer to any perceived perceptual conflict.

**Fig 2:**
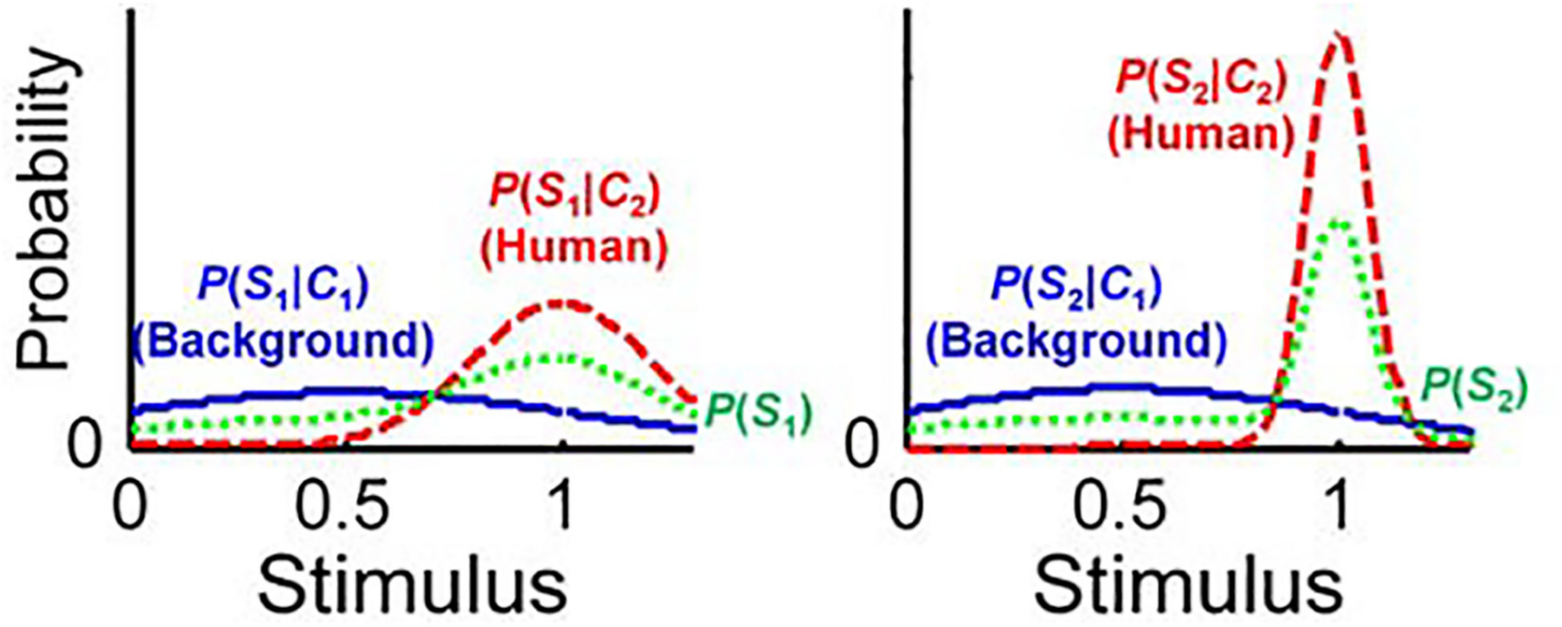
Probability of the occurrence of a different stimulus given a broad human category (*S*_1_), and a narrow human category (*S*_2_). The left- and right-hand panels show the probability densities for *S*_1_ and *S*_2_, respectively.

In this study, we assumed that the perceived stimuli involved two types of stimulus (*S*_1_ and *S*_2_) and two categories (*C*_1_ and *C*_2_), with *P*(*C*_1_) = *P*(*C*_2_) = 0.5. The stimuli *S*_1_ and *S*_2_ have different measures of uncertainty, and the parameters are set to *σ*_s1_ = 0.2 and *σ*_s2_ = 0.05. The categories *C*_1_ and *C*_2_ correspond to the background and human categories. The background category (*C*_1_) parameters are set to *μ*_c1_ = 0.5 and *σ*_c1_ = 0.5, and the human category (*C*_2_) parameters are set to *μ*_c2_ = 1.0 and *σ*_c2_ = 0.05 where the mean of the human category is adjusted to reproduce the uncanny valley effect in ASD. Hence, the posterior probability of the human category given the stimulus *S*_1_ (*P*[*C*_2_ | *S*_1_]), is distributed more broadly and, if given the stimulus *S*_2_ (*P*[*C*_2_ | *S*_2_]) with a narrower distribution, the effect is small on the posterior probabilities of the background category (*P*[*C*_1_ | *S*_1_] and *P*[*C*_1_ | *S*_2_]) (Fig 2). Here, we compute the whole emotional response function *Y*(*S*_1_, *S*_2_) as the linear sum of the emotional responses induced by the stimuli *S*_1_ and *S*_2_. The whole emotional response is given by:

$$Y(S_{1},S_{2})=\beta \cdot F(S_{1})+(1-\beta )\cdot F(S_{2}),$$ (6)

where *β* is the attention rate for the stimulus *S*_1_, and is set to 0.5.

### The uncanny valley effect in ASD

**Fig 3:**
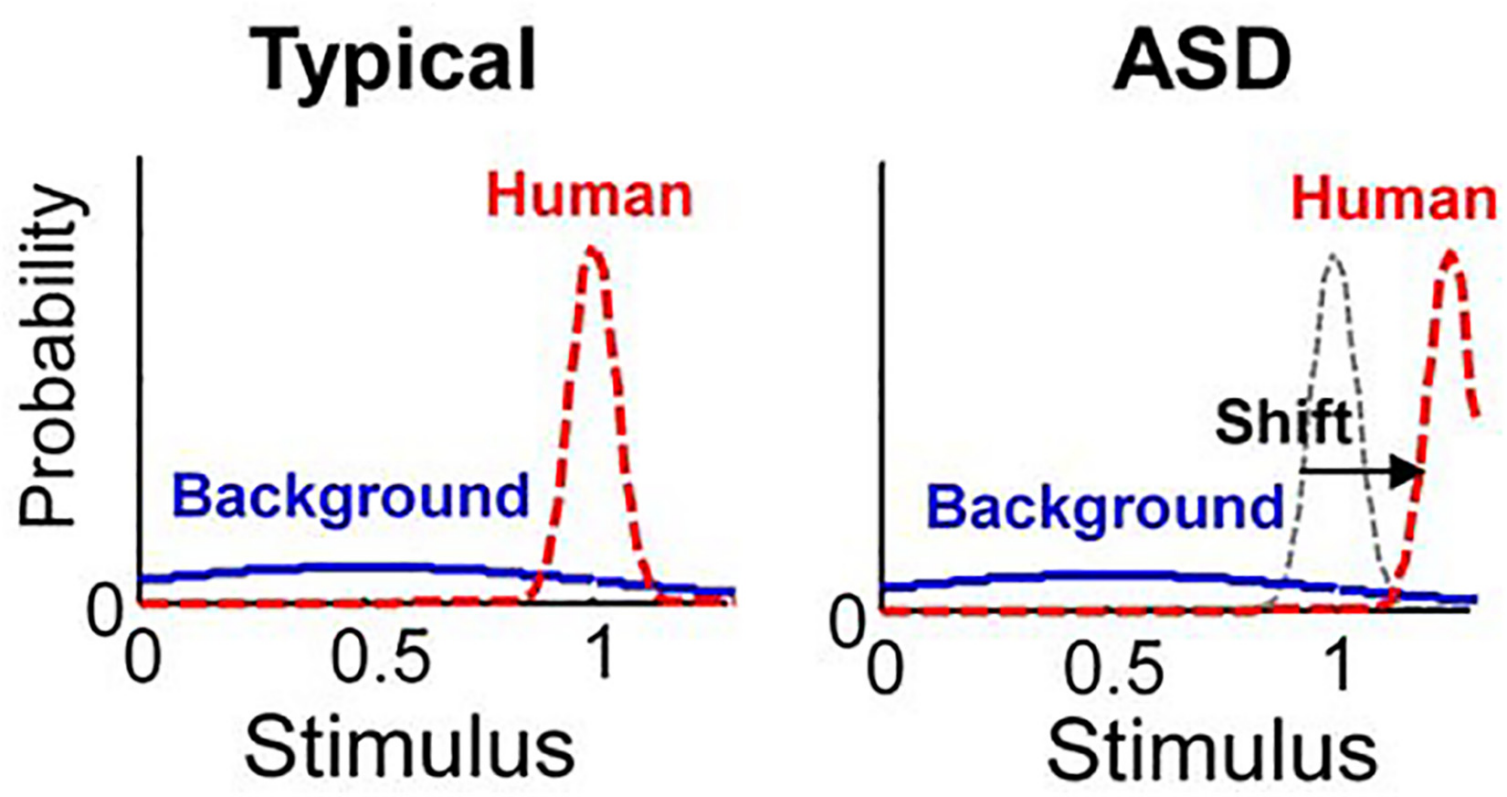
Probability densities of perceptual categories. The typical and ASD conditions are defined by *μ*_c2_, where the mean of the human category is: typical, *μ*_c2_ = 1; ASD, *μ*_c2_ = 1.25.

We assumed that ASD involves not the “uncanny valley” but the “uncanny cliff” (Fig 1) because children with ASD have been shown to have persistent deficits in social communication and social interaction with other people, while a realistic human-looking robot did not elicit negative feelings [20, 21]. Hence, although they interact relatively easily with non-human animals and robots, individuals with ASD may have a reduced perception of agency in humans [31]. Additionally, individuals with ASD may perceive less stimulus information when they interact with other persons [23]. We thus hypothesized that individuals with ASD expect people to be even more human-like than they actually are, and that the human category in ASD is shifted from its actual position to an extreme position (Fig 3). Studies using fMRI found that individuals with ASD identify humans utilizing different neural systems, as compared with typical individuals. Individuals with ASD showed either abnormally weak or no activation in the fusiform face area, which is specialized for facial recognition among typical individuals [22], and abnormal functional neural connectivity during face processing [24]. In this study, we reproduced the uncanny valley effects in ASD as the “uncanny cliff” using values for *μ*_c2_ ranging from 1.0 to 1.3.

### Learning process via robot-assisted therapy

Robot-assisted therapies may still help individuals with ASD improve social interaction with other people, because feelings of uncanniness are tied to perceptions of experience [32]. It has been suggested that all of our perceptual categories are inborn [33]. However, the boundaries of inborn categories may be modified as a result of learning [34]. There are two different learning processes: categorical expansion and categorical compression [35]. Because the widths of the boundaries are determined by the variance of the categories, we assumed that the variance of the human category could be updated by experiences of perceived humanness via interactions with therapeutic robots.

We performed simple simulations of robot-assisted therapy based on the assumption that the variance of the human category updates with each trial. In the simulations, the variance of the human category is updated iteratively as follows, in accordance with the learning rule of CP (see Appendix):

$${\left(\sigma_{c2}^{\left(k+1\right)}\right)}^{2}={\left(\sigma_{c2}^{\left(k\right)}\right)}^{2}+\gamma \cdot P(C_{2}|S_{p})\cdot \left\{{\left(S_{p}-\mu_{c2}\right)}^{2}-{\left(\sigma_{c2}^{\left(k\right)}\right)}^{2}\right\},$$ (7)

where *k* and *S*_*p*_ are the trial number and presented stimulus for learning, respectively. Here, the learning parameter *γ* is a variable parameter that is proportional to the emotional response:

$$\gamma =\delta \cdot Y(S_{1},S_{2}),$$ (8)

where *δ* is the scaling parameter, and is set to 0.1. We assumed that the learning process must be dependent on the emotional response *Y*(*S*_1_, *S*_2_), e.g., an interest or motivation. Then, if *Y*(*S*_1_, *S*_2_) becomes positive, it fosters learning. On the other hand, if *Y*(*S*_1_, *S*_2_) becomes negative, it inhibits learning.

The posterior probability *P*(*C*_2_ | *S*_*p*_) represents an effect of the presented stimulus *S*_*p*_ on the perceptual human category *C*_2_. In this study, we defined *P*(*C*_2_ | *S*_*p*_) as the linear sum of the effects of stimuli *S*_1_ and *S*_2_, similar to Eq (6):

$$P(C_{2}|S_{p})=\beta \cdot P(C_{2}|S_{1})+(1-\beta )\cdot P(C_{2}|S_{2}).$$ (9)

## Results

### The uncanny valley model

**Fig 4:**
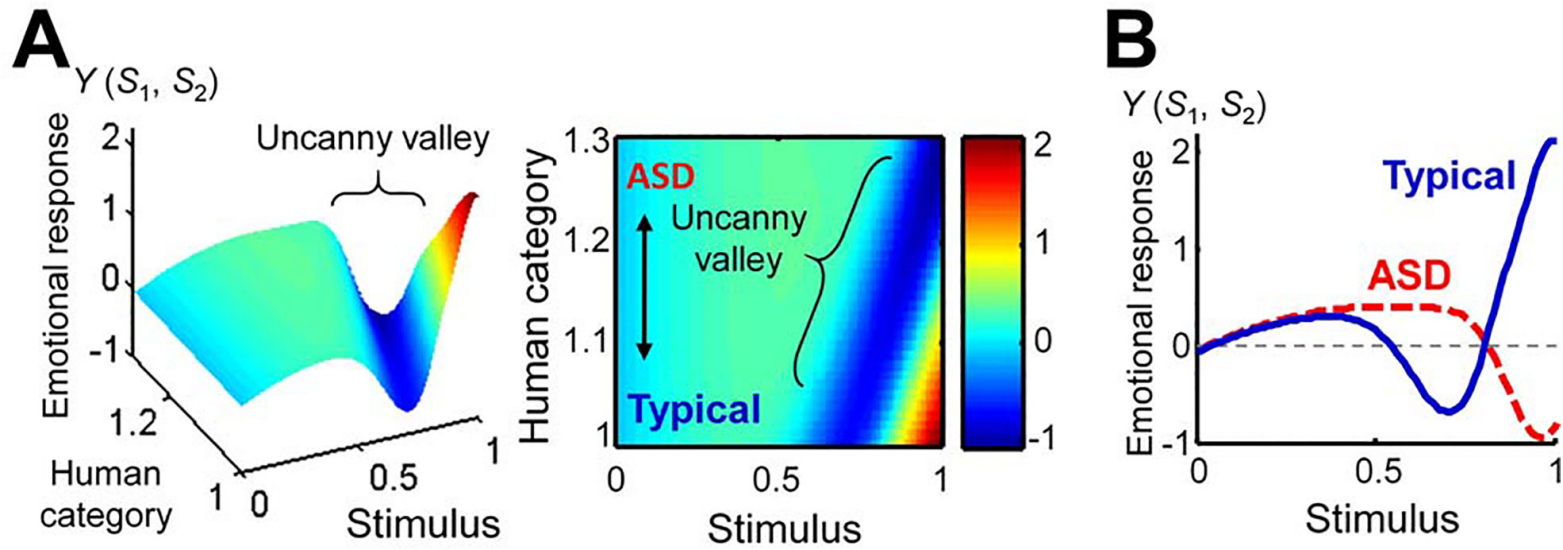
Predictions of the uncanny valley effects according to Moore’s model. (A) Emotional response plotted as a function of the stimulus and the mean of the human category. The left- and right-hand panels show the three-dimensional representation, and the color image of a plain face, respectively. (B) The uncanny valley curves for the typical and ASD conditions (typical, *μ*_c2_ = 1; ASD, *μ*_c2_ = 1.25).

We reproduced the uncanny valley effect as an emotional response to stimuli using a Bayesian model of the uncanny valley effect. We also tried to predict autistic behavior by adapting the same model using a shifted human category for the perception of humanness. When the mean of the human category was shifted to the right, the uncanny valley receded (Fig 4A) and the reduced emotional response could not be recovered at the upper bound of the stimulus. The uncanny curve formed a “cliff” rather than a “valley”, in keeping with our original assumption (Fig 4B).

### Effects of robot-assisted therapy

**Fig 5:**
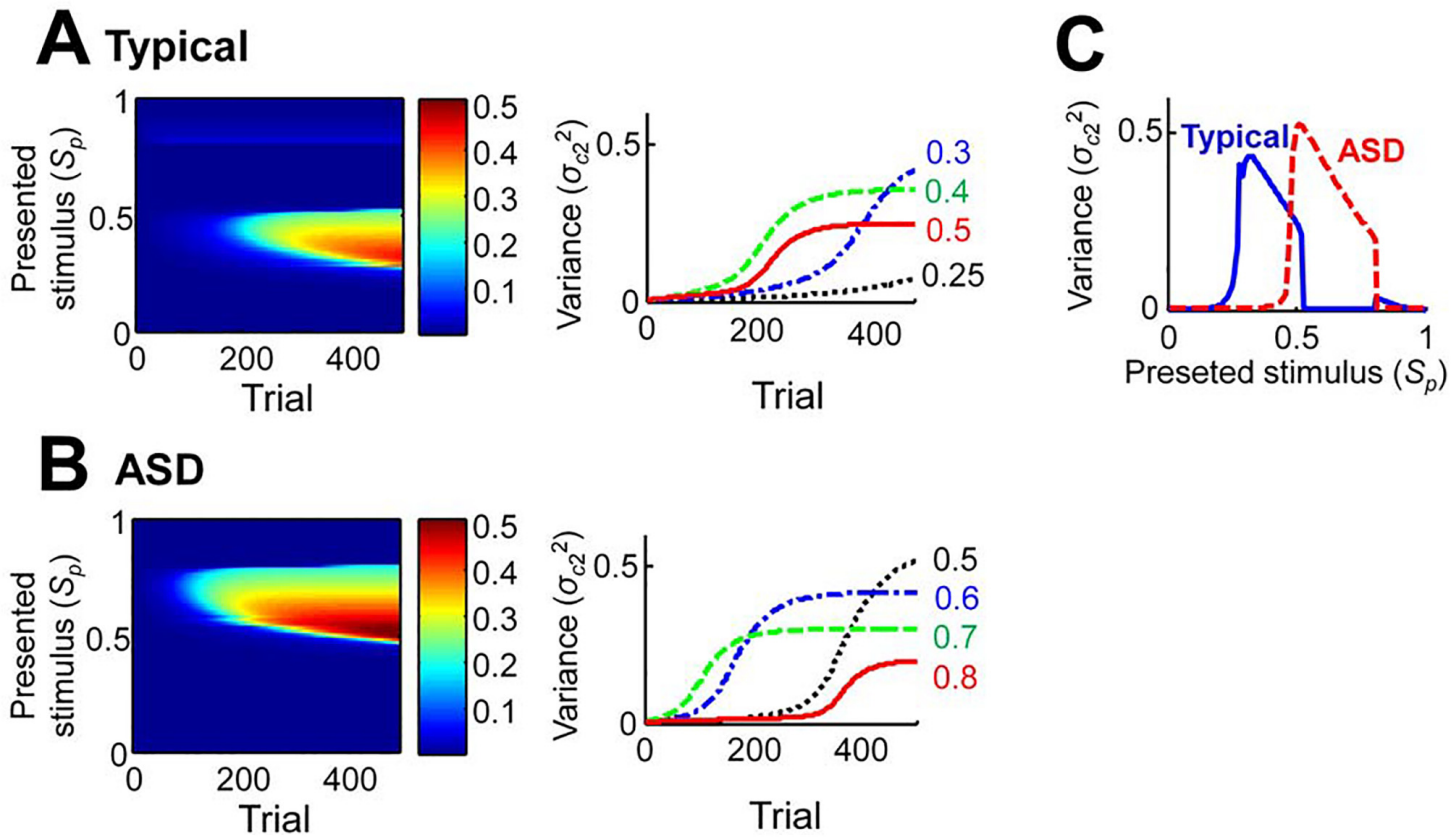
Effects of robot-assisted therapy on the variance of the human category. (A–B) Learned variance of the human category. The left panel is plotted as a function of the trial number and the presented stimulus. The right panel represents the learning curves for some of the presented stimuli: (A) in the typical condition; and (B) in the ASD condition. (C) The terminal values of the learned variance after 500 trials with respect to the presented stimulus.

We simulated processes that numerically evaluated CP learning of a human category under several stimulus conditions to examine the effects of robot-assisted therapy. The value of *μ*_c2_ was set to 1.25 for the ASD condition. The presented stimuli were defined as visual impressions of the therapeutic robots, and were distributed over a range from 0 (completely machine-like) to 1 (fully human-like). The human category was influenced by stimuli between the range of 0.25 and 0.5 in the typical condition (Fig 5A), versus 0.5 and 0.8 in the ASD condition (Fig 5B). The learning speed varied depending on the presented stimulus. The learned variances after 500 trials differed according to the presented stimulus (Fig 5C). In both conditions, the presented stimulus induced a high variance at the lower bound of effective area, and a small variance at the upper bound.

**Fig 6:**
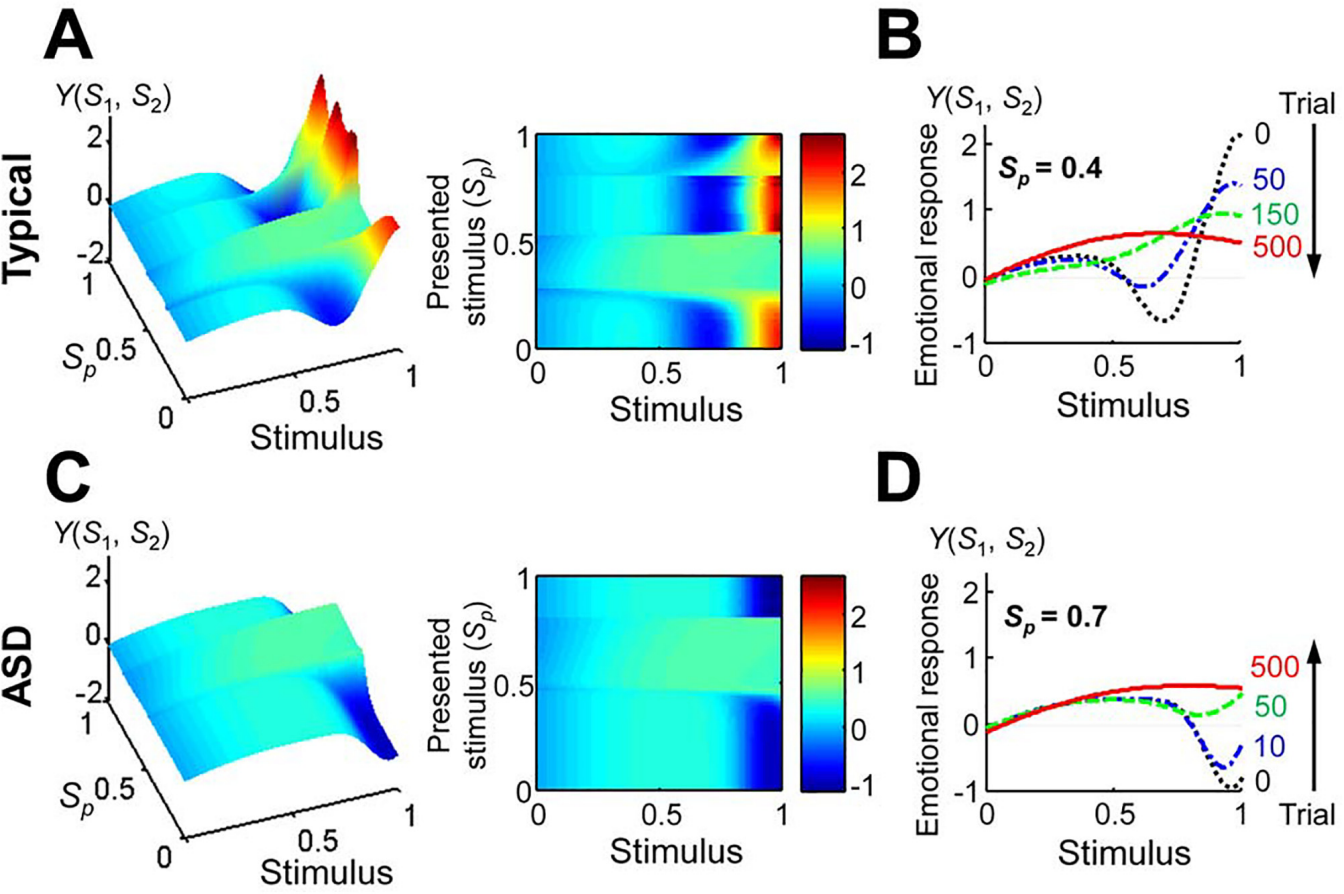
Effects of robot-assisted therapy on emotional response. (A) Emotional response in the typical condition plotted as a function of the stimulus and presented stimulus. The left and right panels correspond to the three-dimensional representation, and a color image of a plain face, respectively. (B) Example of the uncanny valley curve changes in the typical condition for 0.4 of the presented stimulus at 0, 50, 150, and 500 trials. (C) Emotional response in the ASD condition plotted as a function of the stimulus and presented stimulus. The left and right panels correspond to the three-dimensional representation, and a color image of a plain face, respectively. (D) Example of the uncanny curve changes in the ASD condition for 0.7 of the presented stimulus at 0, 10, 50, and 500 trials.

We produced the uncanny valley effect using the learned variances after 500 trials. In the typical condition, the uncanny valley disappeared when the presented stimulus was within a range of 0.25 and 0.5 (Fig 6A). Then the dip was gradually lifted in succeeding trials, and the peak for the human-like stimuli was decreased (Fig 6B). In the ASD condition, the uncanny cliff reappeared when the presented stimulus was within a range of 0.5 and 0.8 (Fig 6C), and the bottom gradually increased across subsequent trials (Fig 6D).

## Discussion

We presented an emotional model of ASD that adapts a Bayesian model of the uncanny valley effect [25]. We then postulated that children with ASD show a unique emotional response curve to human likenesses, forming a cliff rather than a valley. The expected emotional response for individuals with ASD was reproduced by our model. In our study, we carried out numerical simulations of robot-assisted therapy to examine the effects on emotional response. In the typical condition, the simulation showed disappearance of the valley when the presented stimulus was within a certain range (Fig 6A). This result may indicate a habituation effect, because people’s cultural backgrounds may have a considerable influence on the uncanny valley [36], e.g., people who are more accustomed to computer graphics and robots may be less affected by the human likeness of the stimulus. On the other hand, although a stimulus fell into the uncanny valley in the typical condition, it was effective at avoiding the uncanny cliff in the ASD condition. Thus, individuals with ASD may find it more comfortable, and may modify their emotional response, if the robots look like deformed humans, even if they appear creepy to individuals without the disorder. We therefore suggest that robot-assisted therapy using human-looking robots has potential advantages for improving social interaction in individuals with ASD. Because this study has only been conducted using a computational approach, there are limitations to our conclusions for predicting actual human behaviors. Thus, it is necessary to establish the uncanny valley theory in terms of the neural basis, and evaluate our assumptions about the uncanny valley effect perceived by individuals with ASD.

In our simulations, the learned variances differed depending on the presented stimulus (Fig 5). However, the influences were small in terms of the uncanny valley effect (Fig 6). Thus, the response to the presented stimulus was primarily related to the efficiency of the therapy whenever it was within the effective interval.

There are few empirical data supporting the uncanny valley theory, and opinions vary as to the degree of its effect and longevity [37–39]. No empirical studies have directly investigated if and how the uncanny valley applies to those with ASD. Thus, the uncanny valley theory must not be considered as an established theory. On the other hand, several studies clearly suggest that the uncanny valley exists, and it is supposed that the effect is likely to be a great deal more complex than Mori’s original proposal, driven by a number of factors.

The greatest differences in brain responses to uncanny robots were bilateral in the parietal cortex, specifically in the areas that connect the part of the visual cortex that processes body movements with the motor cortex [16]. We therefore postulate that visual feedback systems have developed as systems for classifying self (human-likeness) and others (non-humanness), estimating the internal state of others, and predicting their emotional responses, from a system based on identifying one’s own movements. Visual feedback systems are thought to make a major contribution to social functions. This suggests that the design of therapeutic devices for children with ASD should be carefully considered.

This study proposes a hypothesis about human-robot interaction for individuals with ASD. It may explain the influence of robot-assisted therapy on children with ASD, one that aims to improve their social interactions. We speculate that such therapy induces adaptation of perceptual categories and, consequently, modifies emotional response curves generated by the uncanny valley effect. Moreover, human-looking therapeutic robots, which fall into the uncanny valley in typical individuals, may improve social interaction in individuals with ASD. In the future, we will evaluate our assumption regarding the uncanny valley effect in children with ASD. First, we will measure observers’ impressions of facial images in children with ASD. In doing so, the degrees of realism of the images will be manipulated by morphing between artificial and actual human faces, similar to a previous study in healthy subjects [40]. After that, we will investigate the effects of robot-assisted therapy, thorough human-robot interaction games, relating to physiological indicators (e.g., heart rates [20]) or emotional expressions (e.g., smiles [41]), and specific assessment scales including social interactions for individuals with ASD, i.e., the Social Responsiveness Scale (SRS) and the Autism Diagnostic Observation Schedule (ADOS).

## Appendix

### Derivation of learning rule for categorical perception

Learning processes of CP can be divided into two different processes: learning that occurs between categories and learning that occurs within a category of comparison may be described as a categorical expansion effect and a categorical compression effect, respectively [35]. The categorical expansion effect broadens the category boundaries, allowing the category to encompass a larger set of objects. In contrast, the categorical compression effect narrows the category boundaries to include a smaller set of objects. Because category boundaries are determined by the variance of the categories in accordance with the Bayesian model of CP [29], we supposed that the variance of the category for the stimulus is denoted by:

$$\sigma_{cj}^{2}=E[{\left(S_{i}-\mu_{cj}\right)}^{2}].$$ (A1)

Thus, when a subject is exposed to stimulus *S*_*p*_, the distribution is updated by the stimulus as:

$${\left(\sigma_{cj}^{\prime }\right)}^{2}=(1-P(C_{j}|S_{p}))\cdot \sigma_{cj}^{2}+P(C_{j}|S_{p})\cdot {\left(S_{p}-\mu_{cj}\right)}^{2},$$ (A2)

where *P*(*C*_*j*_ | *S*_*p*_) is the posterior probability and represents an effect of the presented stimulus *S*_*p*_ on the perceptual category *C*_*j*_. From the Eq (A2), the amount of the update can be described as:

$${\left(\sigma_{cj}^{\prime }\right)}^{2}-\sigma_{cj}^{2}=P(C_{j}|S_{p})\cdot \left\{{\left(S_{p}-\mu_{cj}\right)}^{2}-\sigma_{cj}^{2}\right\}.$$ (A3)

Here, we suppose the category variance at the *k* ^th^ trial as ${\left(\sigma_{cj}^{\left(k\right)}\right)}^{2}$. The variance is supposed to be iteratively updated through a driving signal described as Eq (A3). We thus define the learning rule of CP as follows:

$${\left(\sigma_{cj}^{\left(k+1\right)}\right)}^{2}={\left(\sigma_{cj}^{\left(k\right)}\right)}^{2}+\gamma \cdot P(C_{j}|S_{p})\cdot \left\{{\left(S_{p}-\mu_{cj}\right)}^{2}-{\left(\sigma_{cj}^{\left(k\right)}\right)}^{2}\right\},$$ (A4)

where *γ* is the learning parameter.

## Supporting Information

S1 File MATLAB (The MathWorks, USA) code used for all simulations. All relevant data are reproduced by this file.
(M) Click here for additional data file.
